# Case report: Concurrent pylephlebitis and subarachnoid hemorrhage in an octogenarian patient with *Escherichia coli* sepsis

**DOI:** 10.3389/fmed.2023.1158582

**Published:** 2023-05-10

**Authors:** Yong Zhao, Dandan Feng, Xinyu Wang, Yuanyuan Sun, Junni Liu, Xiaodong Li, Nannan Zhou, Jianchun Wang

**Affiliations:** ^1^Department of Geriatrics, Shandong Provincial Hospital Affiliated to Shandong First Medical University, Jinan, Shandong, China; ^2^Department of Geriatric Cardiology, Shandong Provincial Hospital Affiliated to Shandong First Medical University, Jinan, Shandong, China; ^3^Department of Geriatric Neurology, Shandong Provincial Hospital Affiliated to Shandong First Medical University, Jinan, Shandong, China

**Keywords:** sepsis, pylephlebitis, subarachnoid hemorrhage, octogenarian, disseminated intravascular coagulation, anticoagulation

## Abstract

**Background:**

Pylephlebitis refers to an infective suppurative thrombosis that occurs in the portal vein and its branches. Concurrent pylephlebitis and subarachnoid hemorrhage (SAH) are rare but fatal for patients with sepsis. This scenario drives the clinicians into a dilemma of how to deal with coagulation and bleeding simultaneously.

**Case summary:**

An 86-year-old man was admitted to hospital for chills and fever. After admission, he developed headache and abdominal distension. Neck stiffness, Kernig's and Brudzinski's sign were present. Laboratory tests discovered decreased platelet count, elevated inflammatory parameters, aggravated transaminitis, and acute kidney injury. *Escherichia coli* (*E. coli*) were identified in blood culture. Computed tomography (CT) revealed thrombosis in the superior mesenteric vein and portal veins. Lumbar puncture and Brain CT indicated SAH. The patient had eaten cooked oysters prior to illness. It was speculated that the debris from oyster shell might have injured his intestinal mucosa and resulted in bacterial embolus and secondary thrombosis in portal veins. The patient was treated with effective antibiotics, fluid resuscitation, and anticoagulation. The dose titration of low molecular weight heparin (LMWH) under close monitoring attributed to diminution of the thrombosis and absorption of SAH. He recovered and was discharged after 33-day treatment. One-year follow-up indicated that the post-discharge course was uneventful.

**Conclusion:**

This report describes a case of an octogenarian with *E. coli* septicemia who survived from concurrent pylephlebitis and SAH along with multiple organ dysfunction syndrome. For such patients with life-threatening complications, even in the acute stage of SAH, decisive employment of LMWH is essential to resolve thrombosis and confers a favorable prognosis.

## Introduction

Pylephlebitis is a common complication of intra-abdominal infections. The incidence is 0.37–2.7 cases per 100,000 person-years. Most patients (70%) are male with a median age of 50 years. The most common pathogen is *Escherichia coli (E. coli)*, and the mortality rangs from 8.7 to 19%. Sepsis is prevalent in nearly 60% of the patients with pylephlebitis and is a potent risk factor for mortality (1, 2). Sepsis is defined as life-threatening organ dysfunction due to a dysregulated host response to infection (3). The mortality of sepsis is 66.7/100,000 in China, and increases dramatically with age. It is 71.3/100,000 in the elderly aged 60–64 and 3136.5/100,000 in those ≥85 (4). The mortality could conceivably be more horrendous in septic patients who develop disseminated intravascular coagulation (DIC). Here we report a very elderly patient who successfully survived from *E. coli* septicemia complicating DIC, pylephlebitis, subarachnoid hemorrhage (SAH), and reactive arthritis.

## Case description

An 86-year-old man was admitted to our hospital on Feb. 13, 2020 with the chief complaint of chills and fever during the past 13 h. His body temperature had been at the peak of 39.0°C. Apart from fatigue and weakness, he vomited bile-stained fluid. Medical history included well-controlled essential hypertension, stable coronary artery disease, and asymptomatic cholelithiasis. On admission, physical examination did not reveal any remarkable signs. His body temperature was 35.0°C, pulse rate 87 beats per minute, respiratory rate 21 breaths per minute, blood pressure 102/62 mmHg, and oxygen saturation 96%. Electrocardiogram showed sinus rhythm and normal ST segments. Laboratory tests demonstrated that the serum amylase, lipase, and urinary amylase were all within the reference ranges, but the inflammatory parameters increased and the platelet count decreased (102×10^3^/μL). In addition, mild transaminitis and acute kidney injury were present (Table 1). Conventional chest CT, which covered part of the epigastrium, reported not only chronic inflammation in both lungs, but intrahepatic gas and cholecystolithiasis. The gas was perplexing, because it was hard to tell whether it was in the intrahepatic bile ducts or in the portal venous system. Anyway, all the rapid changes indicated a severe infection.

**Table 1:** Laboratory results.

	**Biomarkers**	**Reference**	**Hospitalization**														**Follow-up**			
			**Year 2020**														**Year 2020**			**Year 2021**
			**Feb. 13**	**Feb. 14**	**Feb. 15**	**Feb. 16**	**Feb. 17**	**Feb. 18**	**Feb. 20**	**Feb. 24**	**Feb. 26**	**Feb. 29**	**Mar. 2**	**Mar. 5**	**Mar. 9**	**Mar. 15**	**Apr. 7**	**May. 22**	**Dec. 11**	**Feb. 20**
			**Day 1**	**Day 2**	**Day 3**	**Day 4**	**Day 5**	**Day 6**	**Day 8**	**Day 12**	**Day 14**	**Day 17**	**Day 19**	**Day 22**	**Day 26**	**Day 32**				
Inflammation	WBCs (× 10^3^/μL)	3.5–9.5	5.37	11.56	8.08	7.94	8.56	7.4	11.53	14.08	7.06	7.71	5.29	4.24	5.38	6.81	5.64	6.59	–	5.74
	Neutrophils (%)	40–75	89.7	92.5	85.4	84.3	80	81.4	77.3	88.6	78.6	77.6	68.6	63	64.9	68.3	52.3	49.8	–	48.3
	CRP (mg/L)	0–8	66.16	136.8	138.9	116.8	99.7	60.37	18.55	29.71	24.47	44.3	42.27	24.97	–	5.3	2.14	–	–	–
	PCT (ng/ml)	0–0.05	5.22	10.8	10.99	3.64	1.81	1.25	0.49	0.26	–	0.16	0.14	0.29	0.1	0.06	–	–	–	–
Coagulation	Platelets (× 10^3^/μL)	125–350	102	46	27	60	55	74	179	321	322	311	267	242	234	217	188	182	–	–
	D-dimers (mg/L)	0–0.5	–	>20	>20	7.05	4.41	4.6	6.73	4.35	2.97	2.19	–	1.38	1.2	0.75	0.58	–	–	–
	FDPs (μg/ml)	0–5	–	–	–	–	–	10.67	13.69	8.69	5.95	5.3	–	3.21	3	1.79	2.70	–	–	–
	PT (sec)	9.4–12.5	–	18.5	16	16.7	15.1	12.4	12	12.1	12.4	13.4	–	12.1	12.7	11.8	13.4	12.1	–	–
	Fibrinogen (g/L)	2.38–4.98	–	3.42	3.73	4.04	4.18	3.68	3.02	3.83	3.38	4.09	–	4.14	3.52	3.17	3.31	2.84	–	–
Heart	HS-TnT (pg/ml)	0–14	32.23	112	–	–	22.8	15.48	12.2	10.7	–	–	–	–	–	–	–	–	–	–
	CK-MB (ng/ml)	0.1−4.94	1.16	3.92	–	–	1.07	0.89	0.56	1.17	–	–	–	–	–	–	–	–	–	–
	NT-proBNP (pg/ml)	< 1,800	246.9	–	–	4132	5006	6270	1269	478	–	–	–	–	–	–	–	–	–	–
Liver	AST (U/L)	15–40	71	77	54	39	–	20	61	57	89	68	46	35	23	24	45	35	41	31
	ALT (U/L)	9–50	44	43	33	30	–	19	43	74	103	100	75	51	27	25	49	25	29	23
	GGT (U/L)	10–60	59	84	70	91	–	131	236	398	393	478	432	342	281	189	114	119	107	78
	Total BIL (μmol/L)	3.5–23.5	44.53	52.3	27.97	21.93	–	18.5	14.06	31.61	22.19	26.34	23.08	11.99	13.04	12.23	19.56	25.26	20.35	22.61
	Direct BIL (μmol/L)	0.5–6.5	17.92	20.36	9.56	7.31	–	5.27	3.96	9.35	6.09	7.39	6.41	3.64	3.57	2.75	3.28	4.34	3.48	3.44
	Indirect BIL (μmol/L)	1–17	26.61	31.94	18.41	14.62	–	12.23	10.1	22.26	16.1	18.95	16.67	8.35	9.47	9.48	16.28	20.92	16.87	19.17
	Albumin (g/L)	40–55	42	36	29.7	29.8	–	33.8	32	38.9	32	33	32.1	30.7	33.3	35.3	42.3	40.9	46.3	44.5
Kidneys	BUN (mmol/L)	2.8–7.14	8.7	7.7	11	4.7	3.8	6	9.4	4.3	4.1	3.8	3.4	4.6	4	4.1	5.3	4.2	5.1	5.0
	Creatinine (μmol/L)	0–135	139.4	101.1	107.9	87.9	72.5	74.4	65.7	49.8	49.9	57.5	61.1	58.8	62.7	56.8	73.8	82.6	91.0	81.4
	Cystatin C (mg/L)	0.63–1.25	1.31	1.12	1.22	0.92	0.95	1.08	1.31	0.95	0.99	1.06	0.94	1.07	1.14	1.22	1.24	–	1.26	–
	eGFR (ml/min/1.73m^2^)	>90	44	60	54	75	79	72	64	86	83	77	83	76	72	70	84	–	66	–

ALT, indicates alanine aminotransferase; AST, aspartate aminotransferase; BIL, bilirubin; BUN, blood urea nitrogen; CK-MB, creatine kinase-MB; CRP, C-reactive protein. eGFR, estimated glomerular filtration rate; FDPs, fibrinogen degradation products; GGT, gamma-glutamyl transferase; HS-TnT, high-sensitivity troponin T; NT-proBNP, N-terminal B-type natriuretic peptide; PCT, Procalcitonin; PT, prothrombin time; WBCs, white blood cells.

**Figure 1:**
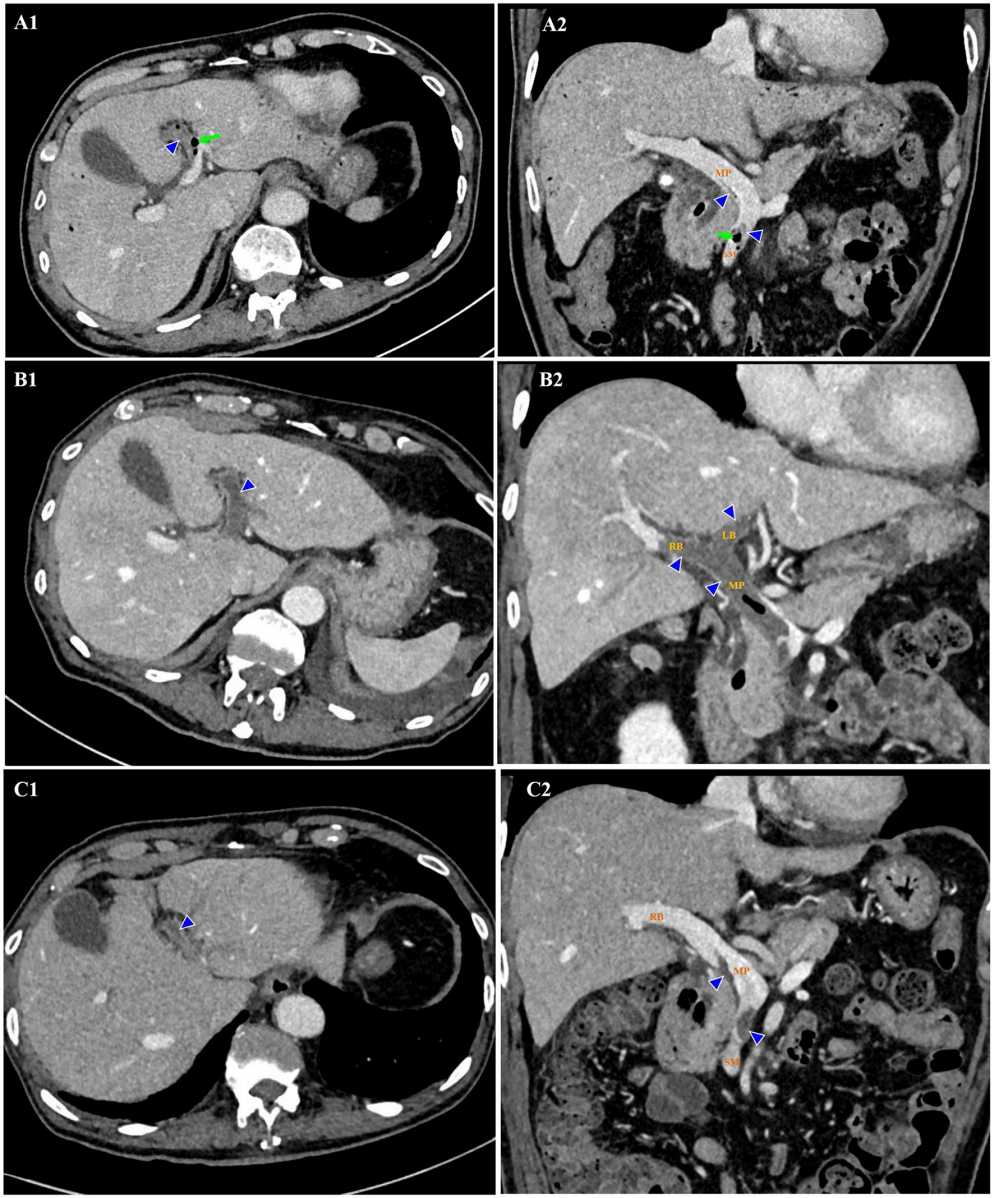
Thrombosis (triangles) and gas (arrows) in the superior mesenteric and portal veins on contrast-enhanced CT. Hospital day 2 (February 14, 2020). Thrombosis and gas in the LB of portal vein on axial view **(A1)**. Thrombosis in the SM and MP, and gas in the SM on coronal view **(A2)**. Hospital day 12 (February 24, 2020). Thrombosis enlarged in the extended LB of portal vein on axial view **(B1)**. Thrombosis enlarged in the MP, LB, and RB of portal vein, with gas in the MP on coronal view **(B2)**. Hospital day 23 (March 6, 2020). Thrombosis shrank in the LB of portal vein on axial view **(C1)**. Thrombosis shrank in the SM, MP, and RB of portal vein on coronal view **(C2)**. No gas was detected. CT indicates computed tomography; LB, left branch (of portal vein); MP, main portal vein; RB, right branch (of portal vein); SM, superior mesenteric vein.

The patient was managed empirically with intravenous moxifloxacin, a fluoroquinolone antibiotic, but his condition deteriorated 8 h after admission. Fever recurred with chills. The temperature increased to 38.8°C. Venous blood was drawn immediately for germiculture and antibiotic susceptibility test. Then, he started to show unbearable headache and abdominal distension, neck stiffness, and Kernig's and Brudzinski's sign. No tenderness was detected in the abdomen. The platelets dropped abruptly to 46×10^3^/μL in 24 h and to 27×10^3^/μL in 48 h. D-dimer was >20 mg/L. Blood culture for 12 h was astonishingly positive for Gram-negative bacilli, and then E. coli was identified. Contrast-enhanced abdomen CT (Figures 1A1, A2) was conducted on hospital day 2. The image of thrombosis with diffuse gas was demonstrated in the superior mesenteric and portal veins, which suggested a hypercoagulable state of DIC caused by *E. coli* bacteremia. Abdominal ultrasonography revealed a normal gallbladder wall and common bile duct, but a mural thrombus in the main portal vein. A trace-back inquiry revealed that the patient had eaten cooked oysters and spat out debris of the shells prior to becoming ill. It was speculated that the debris from oyster shell might have injured his intestinal mucosa and resulted in systemic inflammation and bacterial embolus.

However, the most worrying point was what actually caused the neurological anomaly: purulent meningitis or cerebral hemorrhage? For the time being, the patient was in the hypercoagulable state of DIC, which was manifested as diffuse venous thrombosis. Meanwhile, he got caught in a consumed hypocoagulable stage with an apparent drop of platelets. Hence, intracranial hemorrhage was strongly suspected. Unfractionated heparin was intended to be attempted but was abandoned because of the uncertainty of cerebral condition. Nevertheless, antibiotic regimen was switched to cefoperazone/sulbactam and then imipenem/cilastatin upon results of pathogenic culture and susceptibility test, which showed that the strains of E. coli were most susceptible to these antibiotic agents. Levornidazole was added later to reinforce the antimicrobial therapy. Human immunoglobulin, fresh plasma, recombinant human thrombopoietin, and platelets were administered as well. However, the headache was getting even worse. It was urgent to identify the cause of the encephalopathy. On hospital day 5, a stratification of cerebrospinal fluid (CSF) in the lateral ventricles was suspected on brain magnetic resonance imaging. The substratum had the feature of high signal on T1WI, which implied SAH. Lumbar puncture yielded bloody CSF with an opening pressure of 200 mmH_2_O. Hemophagocytosis was observed, but CSF culture was negative for any bacteria. These results ruled out purulent meningitis and confirmed the presence of SAH. Corticosteroids, diuretics, and mannitol were used to lower the elevated intracranial pressure and prevent cerebral edema. On the very night, the patient experienced a grand mal epilepsy lasting for about 10 min. Phenobarbital sodium was injected intramuscularly, and valproate sodium and levetiracetam were taken orally. The temperature began to drop since the adjustment of antibiotics based on culture result, and the inflammatory parameters declined as well. Although persistent, the headache did not aggravate. Neither chills nor epileptic seizure happened again.

**Figure 2:**
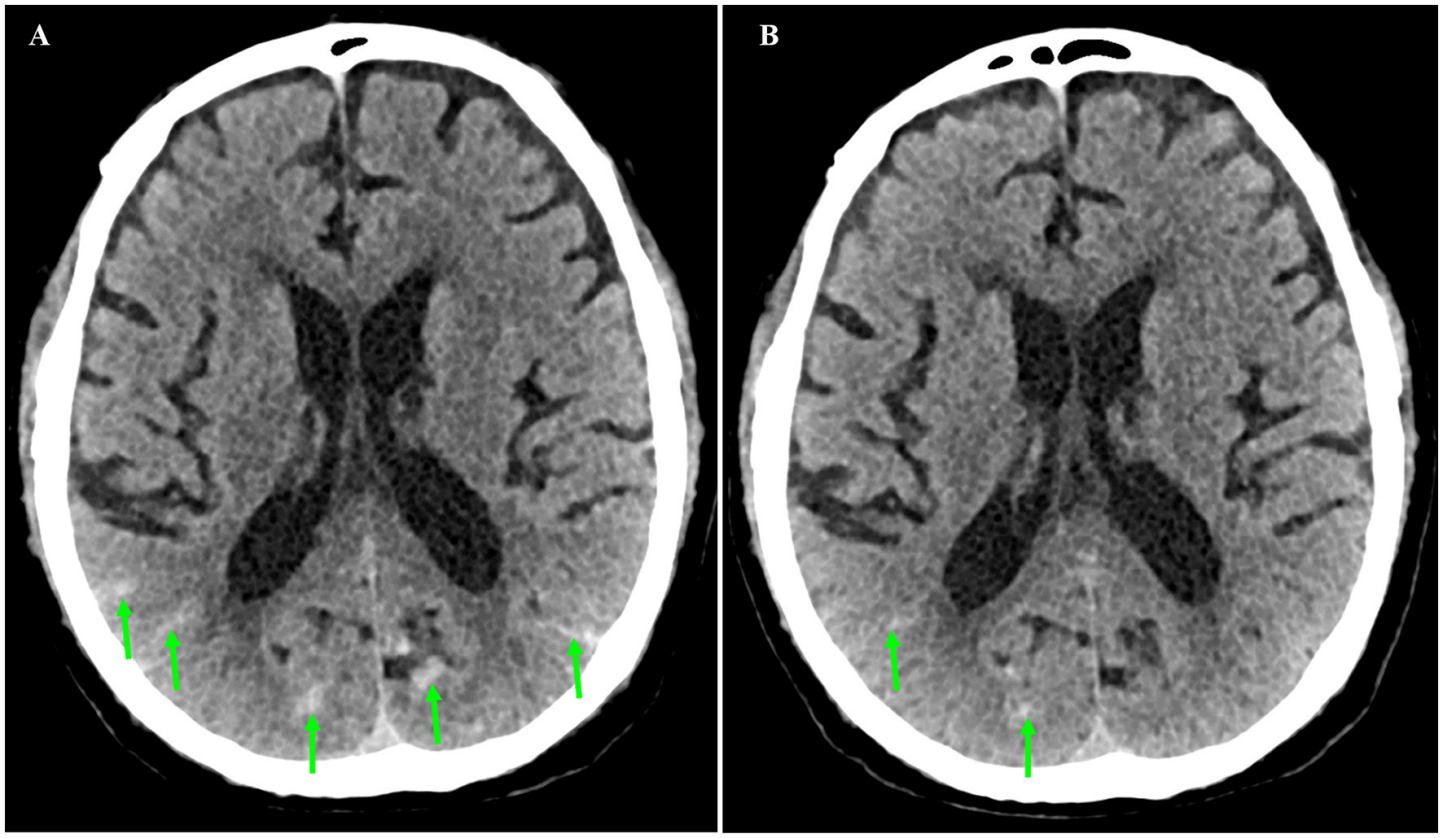
Axial sections of non-contrast brain computed tomography. **(A)** Subarachnoid hemorrhage was present in the right and left parietal and occipital lobes (arrow) on hospital day 12 (February 24, 2020). **(B)** Subarachnoid hemorrhage was almost absorbed on hospital day 23 (March 6, 2020).

It looked like the patient was on the mend, but 10 days after admission, his abdominal discomfort became the dominant symptom. He was anorexic, and complained of abdominal distension. He defecated only once on hospital day 3, and passed less flatus since then. Therefore, adynamic ileus was considered. Meanwhile, the laboratory parameters got worse again. WBC was 14.08×10^3^/μL with 88.6% neutrophilia. Among the hepatic biomarkers, bilirubins were moderately compromised, and gamma-glutamyl transferase (GGT) increased to 398 U/L. Abdominal ultrasonography discovered extension of thrombus from the main portal vein to its branches. Contrast-enhanced abdomen CT was repeated. It showed that the thrombosis in superior mesenteric and portal veins enlarged distinctly, and the left branch of portal vein was completely obstructed, which was speculated to be responsible for above symptoms and aberrant laboratory findings (Figures 1B1, B2). Brain CT demonstrated SAH in the right and left parietal and occipital lobes (Figure 2A).

The patient was caught between Scylla and Charybdis. If the thrombosis had not been treated, liver failure and intestinal necrosis would have been resulted from persistent ischemia. But if anticoagulants had been delivered, SAH might have been exacerbated and threatened his life. We weighted the dilemma carefully. Because the SAH was thought to be diffuse oozing of blood resulting from thrombocytopenia, and the platelet count had recovered to 321×10^3^/μL by now, we decided to try low molecular weight heparin (LMWH) under close monitoring. Enoxaparin was started subcutaneously from a daily small dose of 2000 u. To our surprise, the symptoms and signs of adynamic ileus began to subside on the next day. Enoxaparin was supposed to bring about the improvement. Then it was up-titrated gradually to 3000 u on the 4th day and to 6,000 u on the 6th day. Abdominal ultrasonography detected blood flow in main portal vein, but not in the sagittal part of left portal vein. CT was repeated after 10 days of anticoagulation. In comparison with previous findings, not only thrombosis in the superior mesenteric and portal veins diminished (Figures 1C1, C2), but SAH in both sides was absorbed (Figure 2B). Enoxaparin had been kept in use for 18 days, and then was replaced by oral rivaroxaban.

It seemed that the patient's condition was getting better, however, the adventure was not over yet. When he woke up in the morning of hospital day 19, he felt pain in both wrists, and then fell into a sudden onset of chills with the temperature rising to 38.7°C. Physical examination detected symmetrical redness, swelling, and heat on his wrists. It was considered as reactive arthritis, although his human leukocyte antigen (HLA)-B27 allele was negative. Methylprednisolone and celecoxib were prescribed. This syndrome took a favorable turn on the next day.

After 33-day hospitalization, the patient's condition was greatly improved. Most laboratory abnormalities were normalized (Table 1). He was discharged on March 16, 2020. Table 2 shows the timeline for events and interventions during hospital stay. During the hospitalization, the patient's body weight reduced from 65 to 57 kg. He did not experience a septic shock or fluid depletion. His lowest mean arterial pressure was 72 mmHg, 24-h fluid input was 3,000–4,000 ml, and the urine output was 0.91–3.11 ml/kg/h. Renal function was assessed by estimated glomerular filtration rate (eGFR), which was calculated according to Creatinine-Cystatin C Equation (CKD-EPI 2012) (5). It was improved from 44 ml/min/1.73 m^2^ at admission to 70 ml/min/1.73 m^2^ at discharge.

**Table 2:** Timeline.

**Date (Year 2020)**	**Hospital day**	**Events**	**Interventions**
Feb. 13	1	• Admission to hospital. • Conventional chest CT demonstrated chronic inflammation in both lungs and gas in dilated intrahepatic bile ducts.	• Empiric therapy with intravenous antibiotic: Moxifloxacin.
Feb. 14	2	• Detection of Gram-negative bacilli in blood sample.	• Combination therapy with broad-spectrum antibiotics: Sulperazone and Imipenem. • Administration of intravenous immunoglobulin.
		• Platelet count 46,000/μL.	• Subcutaneous injection of recombinant human thrombopoietin.
		• Contrast-enhanced abdominal CT detected thrombus in the sagittal part of left portal vein and superior mesenteric vein. Gas was found in the main portal vein and its left and right branches, and superior mesenteric vein. But the gas in the left lobe of liver was decreased in comparison with that of last day.	• Supplement with fresh plasma.
Feb. 15	3	• Identification of E. Coli from blood culture.	• Dual antibiotics: Imipenem and Levornidazole.
		• Platelet count 27,000/μL.	• Platelet transfusion.
		• Abdominal ultrasonography revealed mural thrombus in the main portal vein.	• Intravenous infusion of unfractionated heparin.
Feb. 16	4	• Abdominal ultrasonography revealed extension of thrombus from the main portal vein to its sagittal part and superior mesenteric vein.	
Feb. 17	5	• Transient loss of consciousness and epileptic attack. • Subarachnoid hemorrhage confirmed by brain MRI and lumbar puncture.	• Low dose of Enoxaparin, a LMWH, had been tried for once, but then was suspended for fear of major bleeding. • Intracranial pressure was reduced with intravenous Mannitol for 9 days. • Anti-elilepsy was started with oral Depakine for 1 day, and then intramuscular Phenobarbital for 8 days and oral Levetiracetam up to discharge. • Alleviation of cerebral edema with intravenous methylprednisolone for once and then dexamethasone for 6 days. • Prevention of cerebral vasospasm with intravenous Nimodipine for 10 days and then oral Nimodipine up to discharge.
			• Deescalation of antibiotics from combination therapy to a broad-spectrum agent: Meropenem.
Feb. 18	6	• Abdominal ultrasonography detected blood flow in main portal vein, but not in the sagittal part.	
Feb. 20	8	• Blood sample was drawn for the 2nd bacterial culture.	
Feb. 24	12	• Contrast-enhanced abdominal CT demonstrated that the thrombus increased in the portal veins and superior mesenteric vein. The left branch of portal vein was completely obstructed. A small amount of gas was detected in the main portal vein and the lower part of common bile duct.	• Subcutaneous Enoxaparin was administered daily at low dose and then was titrated cautiously.
		• Brain CT confirmed subarachnoid hemorrhage in the parietal, occipital, and temporal lobes of both cerebral hemispheres.	
Feb. 25	13	• No bacteria was isolated in the 2^nd^ blood culture.	• Escalation of antibiotics: Daptomycin was added to Meropenem.
Feb. 26	14	• Abdominal ultrasonography detected blood flow in main portal vein and mesenteric vein, but not in the sagittal part of left portal vein.	
Feb. 28	16	• Blood sample was drawn for the 3^rd^ bacterial culture.	
Mar. 2	19	• Blood sample was drawn for the 4^th^ bacterial culture.	• Deescalation of antibiotic to an effective narrow-spectrum agent: Sulperazone.
		• Reactive arthritis.	• Intravenous Methylprednisolone and oral Celebrex were administered for 4 days, then methylprednisolone was taken orally up to discharge.
Mar. 5	22	• No bacteria was isolated in the 3^rd^ blood culture.	
		• Abdominal ultrasonography detected blood flow in main portal vein, but not in the sagittal part of left portal vein.	
Mar. 6	23	• Contrast-enhanced abdominal CT demonstrated that the thrombus decreased in the portal veins and superior mesenteric vein. But the left branch of portal vein was still completely obstructed. No gas was detected in the main portal vein.	
		• Brain CT demonstrated improvement of subarachnoid hemorrhage.	
Mar. 8	25	• No bacteria was isolated in the 4^th^ blood culture.	
Mar. 13	30		• LMWH and antimicrobial therapy were stopped. Rivaroxaban, a NOAC, was administered.
Mar. 16	33	• Discharge from hospital.	

CT, indicates tomography; LMWH, low molecular weight heparin; MRI, magnetic resonance imaging; NOAC, non-vitamin K antagonist oral anticoagulant.

## Follow-up

Follow-up was performed via face-to-face interview. The post-discharge course was uneventful. Rivaroxaban had been taken orally for 3 months and then was stopped. The patient had no recurrence of pylephlebitis, and engaged in daily activities of life comfortably. He was very grateful to the medical workers for their professional help. Laboratory findings from April, 2020 to February, 2021 indicated that most of the biomarkers in inflammation, coagulation, and hepatic function were within the normal range except for a mildly elevated D-dimers and GGT (Table 1). Abdominal ultrasonography on March 17, 2021 showed that the superior mesenteric vein and the main portal vein were both patent without any thrombosis, but no blood flow was detected in the sagittal part of the left branch of portal vein.

## Discussion

The present case reflects the diagnostic and therapeutic challenge in the elderly with sepsis. Identification of the pathogen and source is quite necessary for the management. Current guidelines recommend that broad-spectrum antibiotics should be administered intravenously within the 1st hour once the diagnosis of sepsis is confirmed (6). In this case, blood culture and antibiotic susceptibility test were performed in time, which was crucial for the subsequent treatment. But it was hard to tell whether the invasive *E. Coli* was from injured intestinal mucosa or chologenic infection. The patient did have cholecystolithiasis, but he vomited bilious contents at the early onset of sepsis, which suggested no obstruction in the biliary tracts. In addition, the gallbladder was not hypertonic, and the location of stones in it was not fixed. It seemed that the chologenic infection was not likely the culprit. On the other hand, the patient came across some debris of shells when he was eating oysters. Thrombosis in the superior mesenteric and portal veins was detected on imaging study, which was probably in part due to bacterial embolus. It was reasonable to think that the injury to intestinal mucosa was the source of sepsis. A review reported that the most common site of thrombosis in patients with pylephlebitis was right portal vein (33%), followed by main portal vein (32%), superior mesenteric vein (31%), left portal vein (24%), splenic vein (18%), and inferior mesenteric vein (8%) (7). For this patient, main portal vein, left portal vein, right portal vein and superior mesenteric vein were all involved. Even anticoagulants were administrated for a long time, thrombosis in the sagittal part of left portal vein was resistant to resolve.

Although pylephlebitis was diagnosed by the fever, bacteremia, and radiological findings of portal and mesenteric vein thrombosis in the patient, it should be differentiated from other causes of acute abdomen presentation with systemic inflammatory response, such as acute suppurative cholecystitis, bacterial hepatic abscess, and acute pancreatitis. In this case, there was no history of chronic hepatic or pancreatic diseases. Physical examination did not find a palpable gallbladder with tenderness in the right upper quadrant of abdomen. Laboratory results showed normal amylase and lipase. Imaging tests did not revealed any signs of emphysematous cholecystitis, hypodense rounded contours in the liver, and enlargement of gallbladder or pancreas. Thus, pylephlebitis was considered as the principal diagnosis for this patient.

In terms of the primary complications of pylephlebites, pyogenic liver abscesses are present in 37% of cases. Other complications include intestinal ischaemia and portal hypertension (1). Multiple organ dysfunction syndrome (MODS) is common in septic patients. Sepsis-associated encephalopathy may result directly from the infection in central nervous system, but more often it is attributable to a variety of sterile neurologic disorders including stroke (8). A post mortem analysis showed that the prevalence of cerebral hemorrhage was 26% in patients who had died from septic shock (9). Lumbar puncture was critical for the differential diagnosis of SAH from purulent meningitis in this patient, and dictated the subsequent strategy of treatment. SAH was attributable to impaired synthesis of coagulation factors by hepatocytes and decreased platelet count because of excessive consumption. Sepsis-associated hepatic dysfunction may result from infection, overactive inflammatory response, or microvascular thrombosis. It is manifested as impaired clearance of bilirubin, decreased synthesis of proteins, disturbance of coagulation, and cholestasis (10). In this case, primary bacterial embolism and secondary thrombosis in the portal veins were responsible for the liver injury. Moreover, the patient had gone through an experience of cardiac and kidney injury. He got a decreased eGFR and elevated cardiac biomarkers (Table 1). Fortunately, these impairments were transient and reversible. He recovered with timely diagnosis and proper treatment.

Sepsis and coagulaopathy are entangled with each other. Thrombocytopenia below 50 × 10^3^/μL strongly suggests an unfavorable prognosis for patients with sepsis (11). International Society on Thrombosis and Haemostasis (ISTH) has devised a composite scoring system for the diagnosis of DIC, and a score equal or more than 5 was adopted as the cutoff value of criteria for overt DIC (12). As for the scoring algorithm criteria established by the Japanese Association for Acute Medicine (JAAM), a total score of 4 was accepted as the cutoff points to diagnose DIC, because it was validated as an early and sensitive predictor of organ dysfunction and poor outcomes in Logistic regression analysis (13). A multicenter retrospective observational study demonstrated that according to ISTH and JAAM criteria, the prevalence of DIC in patients with sepsis on intensive care unit admission was 29% and 61%, respectively (14). However, there is no consensus on the use of anticoagulants in pylephlebitis. It is generally believed that anticoagulation should be delivered to the patients with thrombosis progression. In a retrospective cohort study, 3 points or more according to both the ISTH overt and the JAAM DIC scoring systems was used as the optimal cutoff for initiation of anticoagulant therapy in septic patients with DIC. Results showed that it was associated with a minimal all-cause in-hospital mortality and an acceptable incidence of bleeding complications (15). In addition, for patients with pylephlebitis, those treated with anticoagulants had a higher resolution rate of portal vein thrombosis than non-anticoagulated ones, without significant risk of major bleeding (16). In this case, sepsis-associated coagulopathy was much more complicated. The score was ≥5 points for DIC according to ISTH and JAAM criteria. Widespread thrombosis in the superior mesenteric and portal veins as well as SAH developed successively in only a couple of hours after the onset of sepsis, which was a fatal threat to an 86-year elderly patient. Scrupulous administration of enoxaparin at the right time was of crucial importance for saving his life. The situation did begin to take a change for the better thereafter. All the facts confirmed the benefits of early anticoagulation in septic patients with DIC.

The mortliaty of pylephlebitis ranges from 8.7% to 19%. However, it has been significantly reduced in recent decades due to improvement in the identification and treatment of this disease. The overall mortality was reported to be <10% in patients with pylephlebitis who were diagnosed after 2010 (1). An observational retrospective study from a tertiary hospital in Spain indicated that survivors usually had a good prognosis without recurrence of pylephlebitis, and anticoagulation was associated with a lower mortality (17).

Looking back at this case, we learned that the proper timing for this intervention might be as follows. First, there was no sign of active bleeding, even it was still at the acute stage of SAH. Second, platelet count had recovered to the normal range. Third, bacteremia had been under control. Anticoagulant therapy with LMWH should be started from a small dosage, and then be up-titrated gradually to a larger one under close monitoring.

## Conclusion

We presented herein a rare case of an octogenarian patient with sepsis, who experienced pylephlebitis, SAH, MODS, and reactive arthritis. The pathogen was verified as *E. coli*. This patient had eaten cooked oysters prior to becoming ill. It was speculated that the debris from oyster shell might have injured his intestinal mucosa and resulted in bacterial embolus, which led to a secondary thrombosis in portal veins. Immediate microorganism culture is important for the verification of pathogen and subsequent treatment with optimal antibiotics. With the concurrent hemorrhage and thrombosis, the timing of anticoagulation is supremely important. Even in the acute stage of SAH, decisive employment of LMWH is essential to resolve thrombosis without aggravating bleeding and confers a favorable prognosis.

## Data availability statement

The raw data supporting the conclusions of this article will be made available by the authors, without undue reservation.

## Ethics statement

The studies involving human participants were reviewed and approved by Ethics Committee of Shandong Provincial Hospital Affiliated to Shandong First Medical University. The patients/participants provided their written informed consent to participate in this study. Written informed consent was obtained from the individual(s) for the publication of any potentially identifiable images or data included in this article.

## Author contributions

YZ and JW were responsible for the conception, drafting, and critical revision of the manuscript. DF and XW contributed to the retrieval and review of relevant literature. YS and JL collected and assembled clinical information. NZ analyzed and interpreted the data. XL conducted the follow-up. All authors have read the manuscript and approved it for publication.
